# Involvement of F-Actin in Chaperonin-Containing t-Complex 1 Beta Regulating Mouse Mesangial Cell Functions in a Glucose-Induction Cell Model

**DOI:** 10.1155/2011/565647

**Published:** 2011-11-15

**Authors:** Jin-Shuen Chen, Li-Chien Chang, Chia-Chao Wu, Lai-King Yeung, Yuh-Feng Lin

**Affiliations:** ^1^Division of Nephrology, Department of Internal Medicine, Tri-Service General Hospital, National Defense Medical Center, 325, Section 2, Cheng-Kung Road, Neihu 114, Taipei, Taiwan; ^2^School of Pharmacy, National Defense Medical Center, Taipei, Taiwan; ^3^Department of Medicine, Cardinal Tien Hospital 1, Hsintien, New Taipei City 231, Taiwan; ^4^Division of Nephrology, Department of Internal Medicine, Shuang Ho Hospital, Taipei Medical University, No. 291, Jhongjheng Road, Jhonghe District, New Taipei City 23561, Taiwan

## Abstract

The aim of this study is to investigate the role of chaperonin-containing t-complex polypeptide 1 beta (CCT2) in the regulation of mouse mesangial cell (mMC) contraction, proliferation, and migration with filamentous/globular-(F/G-) actin ratio under high glucose induction. A low CCT2 mMC model induced by treatment of small interference RNA was established. Groups with and without low CCT2 induction examined in normal and high (H) glucose conditions revealed the following major results: (1) low CCT2 or H glucose showed the ability to attenuate F/G-actin ratio; (2) groups with low F/G-actin ratio all showed less cell contraction; (3) suppression of CCT2 may reduce the proliferation and migration which were originally induced by H glucose. In conclusion, CCT2 can be used as a specific regulator for mMC contraction, proliferation, and migration affected by glucose, which mechanism may involve the alteration of F-actin, particularly for cell contraction.

## 1. Introduction

Functions of mesangial cell contraction, migration, and proliferation have been reported to be correlated with the development of diabetic nephropathy (DN). Mesangial cell contraction regulating intraglomerular pressure contributes to the occurrence of glomerular hyperfiltration in early DN and then progresses to end-stage renal disease (ESRD) [1]. Alteration in mesangial cell migration may limit repair following mesangiolysis and thereby contribute to the loss of kidney function in diabetic nephropathy [2, 3]. Aberrant proliferation of mesangial cells is commonly observed in DN that can lead to ESRD [4]. As the integrity of cytoskeletons is changed during mesangial cell contraction, migration, and proliferation, actin, the most important cytoskeletal protein, should play a role in the development of DN [5]. 

Actin is often the most abundant protein in a cell, comprising up to 15% of total protein. The function of actin in eukaryotic cells is ubiquitous, including regulation of cell contraction, adherence, movement, and phagocytosis. Actin in cells is generally present interchangeably between a monomer and a polymer, in which globular-(G-) actin subunits assemble into long filamentous polymers called F-actin. Some cellular functions involving cytoskeletons are regulated by this process of actin polymerization [6]. The chaperonin family may play an important role in maintaining the normal function of actin as the function of chaperone-assisted protein folding in cell allows many cytosolic proteins to attain the correct folded states and functional conformations during protein synthesis or during recovery from their denatured states [7]. 

The two most abundant classes of molecular chaperones are the heat shock protein (HSP)60 and HSP70. HSP60 chaperones, also termed chaperonins, are found in all organisms, and are classified into two distinct types. Type I is discovered in prokaryotic cells and endosymbiotic organelles. Type II is found in eukaryotic cytosol and is termed chaperonin-containing t-complex polypeptide 1 (CCT) [8]. CCT is composed of 8 different subunits (CCT *α* *β* *γ* *δ* *ε* *ζ* *η* *θ*, the equivalent of CCT1, 2, 3, 4, 5, 6, 7, and 8), but their functions are still not well understood. 

Up to present, available data [9–12] suggests that CCT is the only chaperonin known to be abundant in the eukaryotic cytosol [11] and the primary substrate for cytoskeletal proteins, tubulins, and actins. Therefore, it is of interest to know how CCT interacts with cytoskeletal proteins and involves in cell functions, such as contraction, migration, and proliferation. 

Of all CCT subunits, CCT2 has been proven to correlate with the function of actin [9]. Furthermore, CCT2 is suggested to have a relationship with mesangial cell contraction in our own proteomic study [13]. Therefore, in order to investigate the effect of CCT2 on the cytoskeletal functions of mesangial cells, an *in vitro* cell model capable of attenuating CCT2 expression by small interfering RNA (siRNA) was established. The aim of this study is to investigate if a relationship exists between CCT2 and F/G actin on various cell functions, including contraction, migration, and proliferation. 

## 2. Materials and Methods

### 2.1. siRNA Transfection and Experimental Protocol

As our previous study found mouse mesangial cells (mMCs) incubating with high glucose has the upregulation of CCT2, the prime work of this study is to find the optimal amount of siRNA to establish a low CCT2 model. In brief, mycoplasma-free SV 40-transformed mMCs were prepared and kept as described previously [13, 14]. These mMCs (1 × 10^5^/well) were maintained in DMEM+F12 containing 5% FBS and 21.25 mM D-glucose in 6-well plates. Once the cells were subconfluent, the medium was changed to Transfection Medium (Santa Cruz, sc-36868, USA), Transfection Reagent (TR, Santa Cruz, sc-29528, USA), and CCT2 siRNA suspension (Santa Cruz, sc-36625, USA), according to manufacturer's instructions. The volume of TR was fixed at 6 ul per the instructions. The optimal siRNA amount was determined by mMCs transfecting with the different volume of CCT2 siRNA 2, 4, and 6 ul per well. After 12 hrs, the whole medium was changed to 21.25 mM glucose with 1% fetal bovine serum (FBS) for 24 hrs. Cells from these different mixes were harvested for protein extraction. Western blotting was used to analyze the expression of CCT 2. Optimal ratio between the volume of TR and siRNA (TR-to-siRNA) was established. 

Once the optimal ratio of TR-to-siRNA was determined, the subsequent experiments were as follows. First, mesangial cells were treated with TR containing CCT2 siRNA, scramble siRNA (CsiRNA, Santa Cruz, sc-37007, USA), and TR only. After 12 hrs, the concentration of culture medium among the above three groups was changed into 6.6 mM (N) and 21.25 mM (H) D-glucose for another 24 hrs. To validate the sequence specificity of gene knockdown *in vitro*, six groups, TR *p*N, CsiRNA *p*N, CCT2siRNA* p*N, TR *p*H, CsiRNA *p*H, and CCT2siRNA *p*H, were designed for the subsequent experiments assaying the protein expression of CCT2 and cell viability. Due to similar results in groups TR *p*N and CsiRNA *p*N, groups CsiRNA *p*N, CCT2siRNA *p*N, CsiRNA *p*H, and CCT2siRNA *p*H were selected for further experiments concerning the F/G-actin ratio, the arrangement of F-actin, and cell contraction, migration, and proliferation. 

### 2.2. Cell Viability

To test the cell viability, MTT assay was used. Six groups were designed as described above. The MTT method was followed as previously described [14]. 

### 2.3. Western Blot (WB) for the Amount of CCT2 and F/G-Actin Ratio

To determine F/G-actin ratio and CCT2, mMCs were cultured in 10 cm Petri dishes at a density of 1  × 10^6^cells per well for protein extraction. 

For CCT2, in brief, the cells were washed with ice-cold PBS and lysed *in situ* with 0.5 mL ice-cold lysis buffer (2 M thiourea, 7 M urea, 4% CHAPS, and 0.5% ampholyte) at 4°C for 15 mins. For whole-cell preparations, supernatants were collected after centrifugation at 13 000 rpm for 20 mins. Protein concentration was determined using the Bio-Rad protein assay kit (Bio-Rad Hercules, CA, USA). Samples were stored at −70°C until use. The protocol for WB was similar to the method previously described. The membranes were incubated overnight at 4°C with antibodies against CCT2 (Santa Cruz, CA, USA) in a ratio of 1 : 500 in TBST containing 1% BSA. Horseradish Peroxidase-(HRP-) labeled secondary antibody (Jackson ImmunoResearch, PA, USA) was used to detect CCT2 proteins.

For the F/G-actin ratio, protein extracts from mMCs were subjected to an F/G-actin *in vivo* assay kit (Cytoskeleton, CO, USA) based on the manufacturer's protocol. Briefly, cells were lysed with a cell lysis and F-actin stabilization buffer and homogenized using 26G syringes. Cell lysates were centrifuged at 100 000 g for 60 min at 37°C. Then supernatants (G-actin) were separated from the pellets (F-actin) and were immediately placed on ice. Pellets were resuspended to the same volume as the supernatants using ice-cold ddH_2_O containing 1% cytochalasin D and were incubated on ice for 60 mins. Equal amounts of the samples (supernatant and pellet) were loaded into each lane and analyzed by WB with 1 : 500 dilution of antiactin antibody (Cytoskeleton, CO, USA) incubated in a blocking buffer for 1 hr at room temperature (RT). Blots were washed three times, and then the membranes were incubated in a 1 : 10000 dilution of goat anti-rabbit HRP (Jackson Immunoresearch, PA, USA) in TBST for 30 mins at RT. Membranes were washed three times, and the membrane-bound antibody detected was incubated with WB luminol reagent kit (Santa Cruz biotechnology, CA, USA) and captured on X-ray film. 

### 2.4. Immunofluorescence for the Localization of F-Actin

Immunofluorescence (IF) staining of F-actin for cells indicated a cytoskeletal rearrangement in the cells [15, 16]. To localize the expression of F-actin, mMCs were subjected to IF. mMCs were plated on 22 mm glass coverslips, washed with PBS, and then fixed with 3.7% paraformaldehyde for 10 min at RT. Cells were then permeabilized with PBS  + 0.1% Triton X-100 for 5 min at RT, blocked with PBS  + 0.1% BSA for 20 min at RT, then thoroughly rinsed with PBS and stained with Phallacidin conjugate with the green fluorescent Alexa Fluor 488 (Molecular Probes Inc., Cat. No. A12379, Ore, USA). A 1 : 500 dilution in blocking buffer 10 mins was used to label the F-actin. After incubating with phallacidin, the cells were treated with 4′,6-diamidino-2-phenylindole (DAPI, Molecular Probes Inc., Cat. No. D1306, Ore, USA)- dilactate 2 mins to label the nuclei. The developed sections were visualized using an optical photomicroscope (Olympus, Tokyo, Japan). Negative controls, from which primary antibodies were omitted, were included in the assay.

### 2.5. Cell Contraction

Phorbol 12-myristate 13-acetate (PMA, Merck Chemicals Darmstadt, Germany) was used to induce mMC contraction, which was assessed from changes in the planar surface area as described previously [14]. mMCs were cultured in 24-well plates at a density of 1  × 10^3^ cells per well. Cells from study groups for cell contraction were assayed and analyzed. For detecting the role of F-actin, the F/G-actin ratio and the arrangement of F-actin in mMC before and after PMA stimulation were evaluated by WB and IF, respectively. 

### 2.6. Cell Migration

For cell migration, mMCs were cultured in a 6-well plate at a density of 1  × 10^6^cells per well. mMCs from study groups were applied to the assay of cell migration as previously described [17]. At the time point of 0 hr, a single wound was created in the center of the cell monolayer by gentle removal of the attached cells with a sterile plastic pipette tip. The debris was removed by washing with serum-free medium. Subsequently, at the time point of 12 hr, the wound was observed; the cells which migrated into the wounded area or protruded from the border of the wound were visualized and photographed under an inverted microscope. Each experiment was performed at least three times independently. For analysis, an Image J file was opened at 0 and 12 hr and the rate of wound closure was calculated. Data are expressed as percentage wound closure relative to the width of control wounds photographed at 0 hr. The wound closure area of the cells cultured in normal glucose was set at 100% [18]. In addition, mMCs at 0 hr and 12 hr were harvested for determining the F/G-actin ratio and the arrangement of F-actin. 

### 2.7. Cell Proliferation

For the evaluation of cell proliferation, mMCs were cultured in a 96-well plate at a density of 1  × 10^3^cells per well. The study groups were applied to the assay of cell proliferation using MTT assay. The protocol for MTT was performed as previously described [14]. According to our experimental design, cells from study groups were checked with MTT at the time points 0 and 12 hr. At 0 hr, MTT data was analyzed, so the effect of CCT2 siRNA on mesangial cell proliferation was determined. Further, the growth rate of mesangial cell from 0 to 12 hr was calculated. The formula for the growth rate is 12 hr–0 hr/0 hr. Each experiment was performed at least three times independently. In addition, mMCs at 0 and 12 hr were harvested for determining the F/G-actin ratio and the arrangement of F-actin. 

### 2.8. Statistical Analysis

All experiments were repeated at least three times. Results within groups are expressed as the mean  ± SEM. Unpaired *t*-tests were used to assess the statistical significance of differences between two groups, and paired *t*-tests were used for within-group comparisons. ANOVA with Tukey's multiple comparison test was used for comparisons between more than two groups. A *P* value of less than 0.05 was considered significant.

## 3. Results

### 3.1. Optimal Ratio of TR-to-siRNA for Establishing a Low CCT2 Cell Model

**Figure 1:**
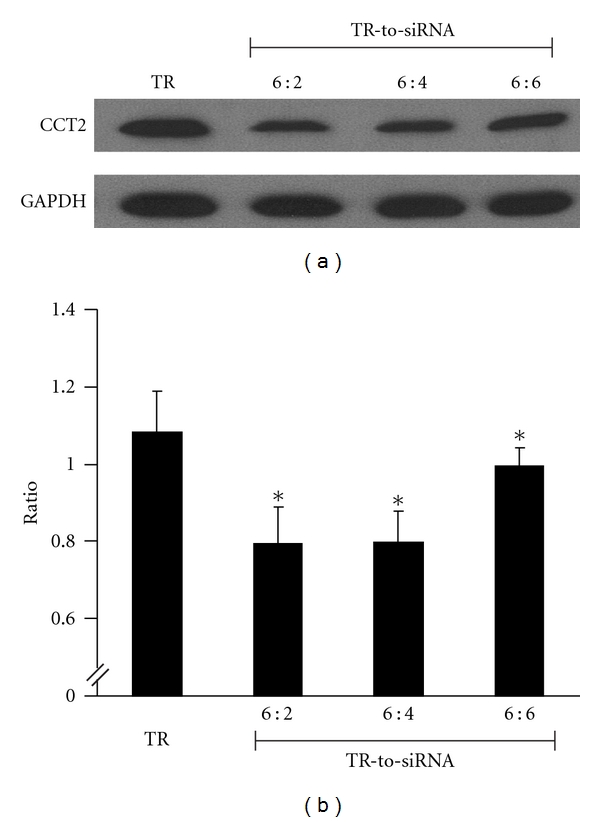
Optimal ratio between transfection reagent (TR) and volume of siRNA (TR-to-siRNA) for a low CCT2 mouse mesangial cell (mMC) model. The volume of TR was fixed at 6 ul, and transfection was performed with varying volumes of CCT2 siRNA. Representative plots show (a) western blotting used for the expression of CCT2 in mMC treated by TR only and TR plus CCT2siRNA at the ratios of 6 : 2, 6 : 4, and 6 : 6 and (b) the normalized CCT2 expression against GAPDH for above groups, in which group 6 : 2 displays an ideal suppressive effect on CCT2 and was chosen for subsequent experiments. *P < 0.05 versus group TR.

As seen in Figures 1(a) and 1(b), groups 6 : 2, 6 : 4, and 6 : 6, all with different volume, showed the attenuating effect on the expression of CCT2 in mMCs when compared to group TR *p*N. The group with the ratio of 6 : 2 showed the most significant effect over the other groups, yielding around 50% reduction of CCT2 level. We suggest that the ideal ratio of TR-to-siRNA is 6 : 2 for blocking CCT2. A low CCT2 cell model is thereby established for subsequent study. 

### 3.2. Cell Viability, CCT2, and F/G-Actin Ratio in Groups with and without Low CCT2 after Glucose Stimulation

**Figure 2:**
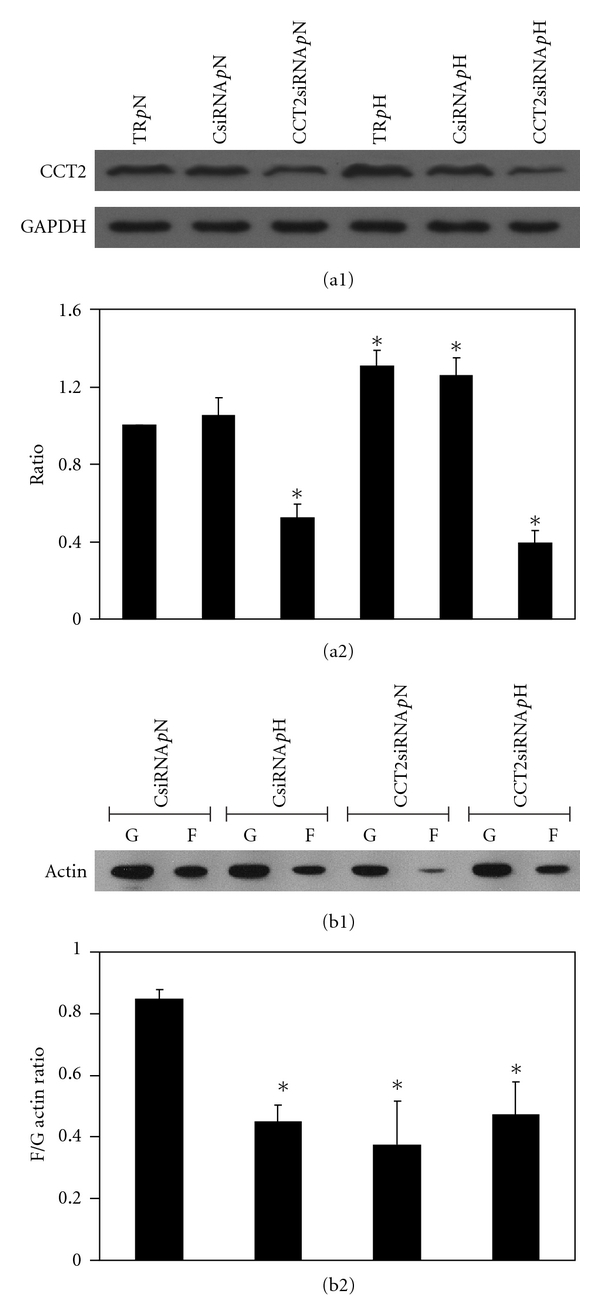
CCT2 and F/G-actin ratios in the CCT2 cell model with and without high glucose induction. The TR-to-siRNA ratio of 6 : 2 was used for the CCT2 knockdown experiment with evaluation of F/G-actin ratio on six designated groups. Representative plots show (a1) western blotting CCT2 expression and (a2) the normalized CCT2 expression against GAPDH for the six groups, in which induction of CCT2 expression was observed with high glucose group of transfection reagent (TR *p*H) and scramble siRNA (CsiRNA *p*H) and reduction of CCT2 expression was with normal glucose group (CCT2siRNA *p*N) and high glucose group (CCT2siRNA *p*H) of siRNA as compared to control group (TR *p*N). *Denotes *P* < 0.05 versus group TR *p*N. Under the same conditions, changes of the F/G-actin ratio were investigated with four subtly designated groups. As representative plot (b1) shows the expression of F- and G-actins responding to the four conditions, and plot (b2) shows the ratios of F-actin and G-actin expressed in the four conditions. Compared to control group CsiRNA *p*N, the decrease of F/G-actin ratio was observed for all experimental groups *P < 0.05.

Using the ratio 6 : 2 of TR-to-siRNA for conducting CCT2 knockdown experiments, six groups, TR *p*N, CsiRNA *p*N, CCT2siRNA *p*N, TR *p*H, CsiRNA *p*H, and CCT2siRNA *p*H, were designed to test the cell viability and to verify the effect of CCT2siRNA. First, there were no differences among six groups for cell viability (data not shown), suggesting that the six groups had a similar outcome for cell viability. Second, as for the expression of CCT2, as Figures 2(a1) and 2(a2) show, mMCs from groups TR *p*H and CsiRNA *p*H both had statistically higher expression than did counter groups TR *p*N and CsiRNA *p*N. There were no differences between TR *p*H and CsiRNA *p*H or between TR *p*N and CsiRNA *p*N, suggesting that scramble siRNA did not lead to degradation of cellular CCT2. Groups CCT2siRNA *p*N and CCT2siRNA *p*H showed a significant attenuating effect when compared to those of other groups. We confirmed that, under the ratio of 6 : 2 between TR and amount of siRNA, the expression of CCT2 decreased significantly in mMCs incubating in normal or high glucose, irrespectively. As groups with and without scramble siRNA showed equal effect on the expression of CCT2, groups CsiRNA *p*N, CCT2 siRNA *p*N, CsiRNA *p*H, and CCT2 siRNA *p*H were chosen for subsequent experiments. 

Protein extracts from four groups, CsiRNA *p*N, CCT2siRNA *p*N, CsiRNA *p*H, and CCT2siRNA *p*H, were investigated for F/G-actin ratio. As Figures 2(b1) and 2(b2) show, the F/G-actin ratio in groups CCT2siRNA *p*N, CsiRNA *p*H, and CCT2siRNA *p*H was statistically lower than that of group CsiRNA *p*N. There was no difference among those three groups, suggesting that high glucose and CCT2siRNA both had a similar effect on mMC attenuating F/G-actin ratio. However, there was no synergetic effect in high glucose plus CCT2siRNA. In summary, our findings suggest that H glucose and low CCT2 may decrease the F/G-actin ratio in mMC.

### 3.3. Change of Cell Contraction, F/G-Actin Ratio, and Arrangement of F-Actin in the Groups with and without Low CCT2 after Glucose Stimulation

**Figure 3:**
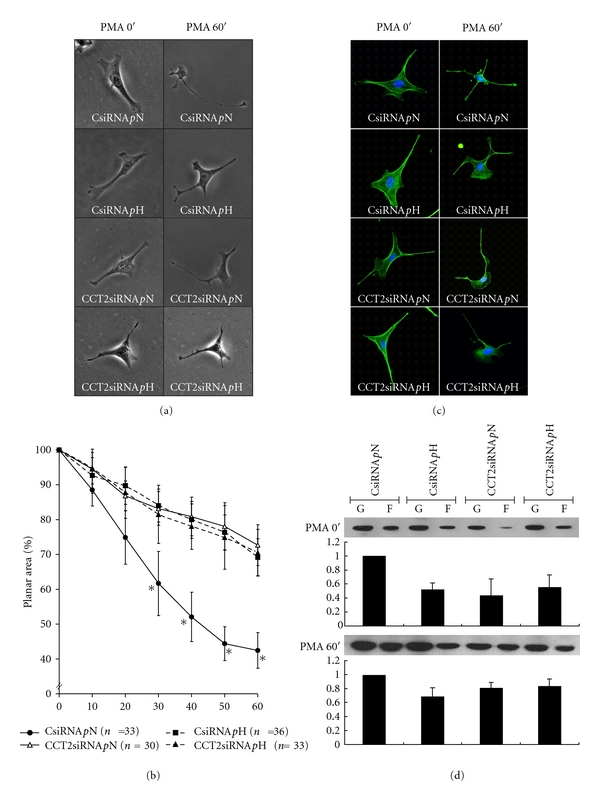
Change of cell contraction, F/G-actin ratio, and arrangement of F-actin in the CCT2 cell model with and without glucose stimulation. Four designated conditions varying in culture medium containing CCT2 or scramble siRNA were used to study the changes of the planar areas of mMC in response to 1 *μ*M PMA stimulation. Representative plots show (a) the morphological changes of mMC (magnification 250x) before (0 min) and after (60 min) PMA treatment and (b) the degrees of cell contraction recorded at 10-min intervals. As group CsiRNA *p*N exhibited the highest contractility with 40–50% planar area reduction at 60 min, the other groups disclosed lower contractility 30 min after PMA stimulation, whereas *n* denotes the number of cells and ∗ represents *P* < 0.05 versus CsiRNA *p*N. Changes in actin were studied in the same conditions. Representative pictures show (c) the immunohistochemical blotting of actin in mMC with highlighting F-actin (green) and nuclei (blue) and (d) the change of F/G-actin ratio before and after PMA stimulation, in which a similar pattern persisted for the four groups.

As shown in Figures 3(a) and 3(b), changes in the planar surface areas of mMCs in response to 1 *μ*M PMA stimulation every 10 min were observed for groups CsiRNA *p*N, CCT2siRNA *p*N, CsiRNA *p*H, and CCT2siRNA *p*H. Differences in the baseline planar areas of these four groups were not statistically significant before the addition of PMA (data not shown). After PMA stimulation, mMC planar areas of the group CsiRNA *p*N decreased to 40%–50% of their original areas. However, groups CsiRNA *p*H, CCT2siRNA *p*N, and *p*H showed the smallest change in planar surface area when compared to that of group CsiRNA *p*N, and no significant difference in contractility was observed among these three groups. Our findings suggest that mMCs treated by H glucose or CCT2siRNA both showed a similar effect on the attenuating of PMA-stimulated contraction, and there was no synergetic effect for H glucose plus CCT2siRNA. 

Taking the results of the expression of CCT2, F/G-actin ratio, and cell contraction together, groups with low CCT2 had an associated low F/G-actin ratio before PMA stimulation, and then these groups displayed significantly less cell contraction than that of normal F/G-actin ratio. 

As for the expression of F/G-actin ratio and the arrangement of F-actin in mMCs before and after PMA stimulation, as Figure 3(c) shows, the change of F-actin distribution in mMC had obviously redistributed from bundle to aggregation after PMA 60′ stimulation. As Figure 3(d) shows, the expression of F/G-actin ratio in all groups remained a similar pattern before and after PMA stimulation. 

In summary, our findings suggest that mMCs with low CCT2 or treated by H glucose may decrease the F/G-actin ratio and also show less cell contraction in PMA-induced cell contraction and F-actin aggregation after PMA, but maintain a similar F/G-actin ratio before and after PMA stimulation. 

### 3.4. Change of Cell Proliferation, Migration, F/G-Actin Ratio, and Arrangement of F-Actin in Groups with and without Low CCT2 after Glucose Stimulation

**Figure 4:**
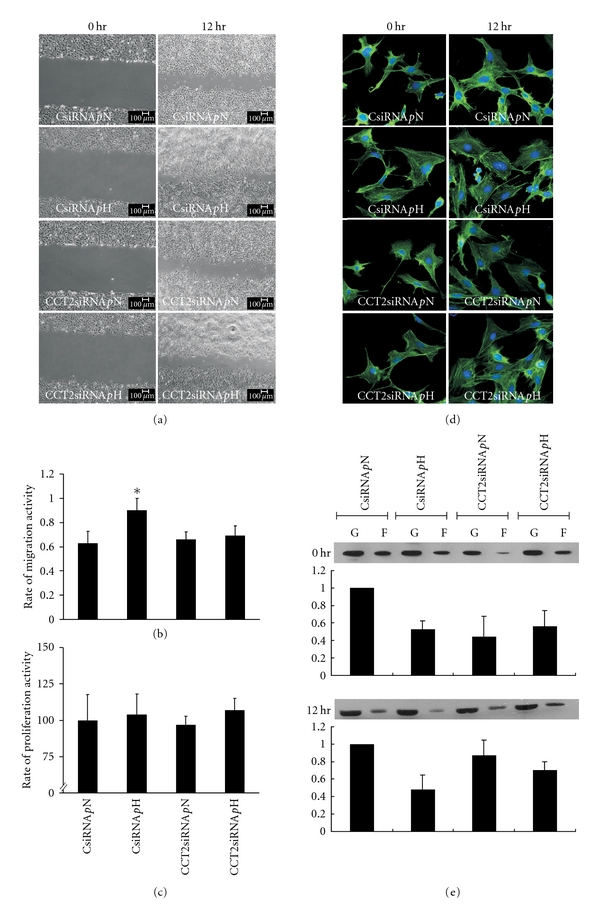
Change of cell proliferation, migration, F/G-actin ratio, and arrangement of F-actin in CCT2 cell model with and without high glucose stimulation. The same four designated groups in previous experiments were studied with high and normal glucose cultured for 24 hrs. Then, wound healing assay was performed to evaluate mMC migration. Representative pictures show (a) the images of mMC migration to close the wound, (b) the rate of migration, and (c) the rate of proliferation during the 12 hr testing interval. Group CsiRNA *p*H exhibited the highest migration activity among these groups. These experiments were performed in triplicate, and the rate of migration activity was calculated as %  = (area at 0 hr–area at 12 hr)/area at 0 hr. Changes in actin were also studied. Representative pictures show (d) the immunohistochemical blotting of actin with highlighting F-actin (green) and nuclei (blue) and (e) the change of F/G-actin ratio during the migration testing interval. As seen, a similar pattern of no significant change was observed across the four groups.

For the change in mesangial cell proliferation under treated conditions, the data of MTT at time points 0 and 12 hr from groups CsiRNA *p*N, CCT2siRNA *p*N, CsiRNA *p*H, and CCT2siRNA *p*H were analyzed. At the point of 0 hr, group CsiRNA *p*H showed a significant increase over that of groups CsiRNA *p*N, CCT2 siRNA *p*N, and *p*H, suggesting that H glucose increased mMCs proliferation, but CCT2 siRNA blocked this effect (data not shown). Furthermore, as Figure 4(c) shows, the rate of mMC proliferation between 0 hr and 12 hr was calculated, and no significant changes among these four groups were found. Taken together, our findings suggest that H glucose increases mMC proliferation, but CCT2 siRNA reverses it. 

For the observation of mMC migration, the percentage of wound closure at the time points 0 and 12 hr from groups CsiRNA *p*N, CCT2 siRNA *p*N, CsiRNA *p*H, and CCT2siRNA *p*H was analyzed. Group CsiRNA *p*H showed significantly enhanced ability of mMC migration when compared to that of groups CsiRNA *p*N, CCT2siRNA *p*N, and* p*H (Figures 4(a) and 4(b)). Among CsiRNA *p*N, CCT2siRNA *p*N, and *p*H, there was no difference in the effect on cell migration, suggesting CCT2siRNA may have a reversing effect, particularly in mMCs incubating in H glucose. 

As for the arrangement of F-actin and expression of the F/G actin ratio in the process of mMC proliferation and migration, the distribution of F-actin and F/G-actin ratio in mMC at 0 and 12 hr showed no significant change (Figures 4(d) and 4(e)). 

Taking the data of migration and proliferation together, our findings suggest that H glucose activates mMC proliferation and enhances the ability of mMC migration, which can be blocked by CCT2siRNA, but is not related to the change of F-actin. 

## 4. Discussion

In our study, a low CCT2 cell model was established, and we found that the ideal ratio of TR-to-siRNA was 6 : 2 for blocking CCT2 in mMC. Second, a novel finding was that mMCs treated by H glucose or with low CCT2 showed less cell contraction induced by PMA, which mechanism involves decreasing F-actin. Third, H glucose activated mMC proliferation and enhanced the ability of mMC migration, which can be blocked by CCT2siRNA, but is not related to the change of F-actin. In summary, CCT2 is a regulator of mMC functions, which mechanisms may involve F-actin, particularly for cell contraction. 

A new low CCT2 mMC model is established by siRNA in our study. The method of siRNA has been used to silence gene expression for studying gene function in cultured cells since 1998 [19]. However, the knockdown efficiency is dependent on the cell line, culture condition, amount of transfection reagent, siRNA quantity and quality, and exposure time of transfection agent to cell. Determination of the transfection efficiency is recommended when using a new cell line. In our study, the ratio of 2 : 6 may not completely block the function of CCT2, but it appears to be adequate for downregulating CCT2 in mMC. 

The relationship between CCT2 and kidney cells has never been studied in the past; our findings firstly demonstrate that CCT2 is a regulator of mMC functions affected by glucose. CCT is a mammalian cytosolic chaperonin required for proper folding of proteins involved in cytoskeletal formation and contractility activity [20–22]. CCT has eight subunits, but the subunits of CCT differ appreciably among cell types [23], and the levels of different CCT subunits may have distinct expression in response to different stresses [24, 25]. Of eight subunits, CCT2 was assigned in this study based on our previous study [13]. We hypothesized that CCT2 would play a major role regulating mMC functions affected by glucose, also taking into account previous findings that CCT2 is related to regulation of cell cycle, neuronal differentiation, and stimulation of chemical stress [23]. The novel finding of our study is that blocking CCT2 in mMC may inhibit cell proliferation, migration, and contraction induced by H glucose stress. 

The relationship between CCT and cell cytoskeleton has been proved by considerable evidence, but there has been no study focusing on CCT or CCT2 and kidney cells. Actin regulating cell movement and division [26] has been known to fold by CCT for 20 years [23, 27–29]. Evidence suggests that disruptions to actin dynamics can lead to changes in cell morphology and cytoskeletal assembly [26, 30]. Of CCT's 8 subunits, individual CCT subunits play a role in maintaining the normal function of actin [26]. Based on our data, knockdown of the expression of CCT2 in mMCs can lead to the significant decrease of F-actin, resulting in the alteration of cell contraction induced by PMA. 

Accordingly, attenuating the expression of CCT2 by siRNA in mMC has a similar effect as that of mMCs treated by H glucose, showing a decrease in F/G actin ratio and the lessening of PMA-induced cell contraction. It is known that cell contraction is modulated by actin polymerization, which involves a number of signal transduction pathways [6]. In this regard, the relationship among H glucose, F-actin, and mMC contraction may be rationalized as follows. In 1998, Zhou et al. have suggested that high glucose alters actin assembly in mesangial cell *in vivo* and *in vitro*, which could account for lack of response to vasopressor agents [31]. Our data was consistent with this observation in mMC. Next, in terms of the relationship among CCT2, F-actin, and mesangial cells, Brackley et al. demonstrated that individual CCT plays a role in the expression of F-actin and then influences cytoskeletal organization [32, 33]. Also, they suggest that CCT monomers have a function independent of the CCT oligomer to indicate that some CCT subunits may carry some forms of chaperone activity. In our study, the real role of CCT2 played by monomers or oligomers was not investigated, but available data strongly suggested that, in mMCs, attenuating the expression of CCT2 could lead to the decrease of F/G actin ratio, subsequently lessening the PMA-induced cell contraction. Furthermore, it was not observed that H glucose plus CCT2 siRNA has a synergetic effect on decreasing cell contraction and F/G actin ratio. As the observation is hard to prove with our data, our hypothesis is that both factors may share the same pathway to influence cell contraction. 

In the study, another novel finding is that CCT2siRNA lessens proliferation and migration in H glucose-induced mMCs. In terms of CCT and cell proliferation, Grantham et al. have demonstrated that siRNA-treated human cell lines with a reduced CCT level show growth arrest [33], and the degree of growth arrest highly correlates with the extent of CCT depletion. However, which CCT subunits respond to cell proliferation is not elucidated. In this regard, in our study, the role of CCT2 in the proliferation of mMC was established. On the other hand, regarding CCT and cell migration, although Satish et al. suggest that downregulating CCT6 blocks the migration of fibroblast induced by epidermal growth factor and platelet derived growth factor [34], our findings show that downregulating CCT2 blocks H glucose-induced mMC migration. Nevertheless, this finding is different from that of Satish et al., suggesting the chaperone activity of individual CCT monomers is not conserved over different cell lines. 

In conclusion, the reduction of CCT2 with siRNA significantly reduced the contraction, proliferation, and migration of mMC, involving the alteration of F-actin, particularly in contraction. CCT2 appears to have a significant biological effect on the process of cellular motion.
